# “For and against” factors influencing participation in personalized breast cancer screening programs: a qualitative systematic review until March 2022

**DOI:** 10.1186/s13690-024-01248-x

**Published:** 2024-02-22

**Authors:** Celmira Laza, Ena Niño de Guzmán, Montserrat Gea, Merideidy Plazas, Margarita Posso, Montserrat Rué, Xavier Castells, Marta Román

**Affiliations:** 1 Department of Nursing and Physiotherapy, University of Lleida, Lleida, Spain; 2 Biomedical Research Institute of Lleida Fundació Dr. Pifarré (IRBLleida), Lleida, Spain; 3 Cancer Prevention and Control Program, Institut Català d’ Oncologia, Barcelona, Spain; 4 Cochrane Associated Center- University Foundation of Health Sciences, Bogotá, Colombia; 5 Department of Epidemiology and Evaluation, Hospital del Mar Research Institute, Barcelona, Spain; 6 Basic Medical Sciences, University of Lleida, Lleida, Spain

**Keywords:** Personalized screening, Breast cancer, Women, Healthcare professionals, Participation, Systematic review

## Abstract

**Background:**

Personalized breast cancer screening is a novel strategy that estimates individual risk based on age, breast density, family history of breast cancer, personal history of benign breast lesions, and polygenic risk. Its goal is to propose personalized early detection recommendations for women in the target population based on their individual risk. Our aim was to synthesize the factors that influence women’s decision to participate in personalized breast cancer screening, from the perspective of women and health care professionals.

**Methods:**

Systematic review of qualitative evidence on factors influencing participation in personalized Breast Cancer Screening. We searched in Medline, Web of science, Scopus, EMBASE, CINAHL and PsycINFO for qualitative and mixed methods studies published up to March 2022. Two reviewers conducted study selection and extracted main findings. We applied the best-fit framework synthesis and adopted the Multilevel influences on the cancer care continuum model for analysis. After organizing initial codes into the seven levels of the selected model, we followed thematic analysis and developed descriptive and analytical themes. We assessed the methodological quality with the Critical Appraisal Skills Program tool.

**Results:**

We identified 18 studies published between 2017 and 2022, conducted in developed countries. Nine studies were focused on women (*n* = 478) and in four studies women had participated in a personalized screening program. Nine studies focused in health care professionals (*n* = 162) and were conducted in primary care and breast cancer screening program settings. Factors influencing women’s decision to participate relate to the women themselves, the type of program (personalized breast cancer screening) and perspective of health care professionals. Factors that determined women participation included persistent beliefs and insufficient knowledge about breast cancer and personalized screening, variable psychological reactions, and negative attitudes towards breast cancer risk estimates. Other factors against participation were insufficient health care professionals knowledge on genetics related to breast cancer and personalized screening process. The factors that were favourable included the women’s perceived benefits for themselves and the positive impact on health systems.

**Conclusion:**

We identified the main factors influencing women’s decisions to participate in personalized breast cancer screening. Factors related to women, were the most relevant negative factors. A future implementation requires improving health literacy for women and health care professionals, as well as raising awareness of the strategy in society.

**Supplementary Information:**

The online version contains supplementary material available at 10.1186/s13690-024-01248-x.

## Introduction

Breast cancer screening by mammography of women in the target population is the main tool for early detection of this disease, and thus reduce mortality from this cause [1, 2]. However, it has been shown that the current strategy based only on age, generally 50 to 69 years of age, has adverse effects that have a negative impact on health systems and women’s lives [3]. Research has focused on moving to a more personalized paradigm that allows preserving and increasing the benefits of early detection (reduction of mortality), reducing the impact of its adverse effects (false-positives, over-diagnosis) [4, 5].

Personalized risk-based screening is a promising strategy that aims to improve on the current strategy by providing earlier detection in women at higher risk and reducing adverse effects in women at lower risk [6]. This involves assessing the risk of each woman, mainly using age, reproductive history, breast density, family history of breast or ovarian cancer, previous benign breast disease, hormonal and lifestyle factors, and a combination of common genetic variants such as single-nucleotide polymorphisms (SNPs) [7]. It also involves stratifying the population into various risk groups, assigning individuals to a specific risk group, and tailoring prevention and early detection interventions to each group [8]. Thus, the aim is to estimate the individual risk of developing breast cancer over a specific time horizon and to provide personalized recommendations for early detection that combine the frequency of screening (annual, biennial, triennial); the starting and ending age of screening; and its modality (mammography, ultrasound and magnetic resonance imaging) [7].

Despite the encouraging of this paradigm, its future implementation faces major organizational challenges, and its success depends on the acceptance of stakeholders, but also of invited women and health care professionals (HCPs) [9]. For this reason, the recommendations of the European Collaborative on Personalized Early Detection and Prevention of Breast Cancer (ENVISION) promote multidisciplinary research on the implementation of personalized screening in real settings, involving all stakeholders. They also encourage that this process should be assessed in each setting and in line with the readiness of healthcare organizations for change, and the values, preferences and social norms [6].

To provide answers to the above mentioned, the WISDOM and MyPeBS clinical trials are currently underway, as well as the PROCAS, BC-PREDICT, PERSPECTIVE and PRISMA prospective cohorts. These studies have not only advanced in the generation and validation of screening strategies based on women’s individual risk, but also in identifying the key factors to be considered for future implementation, from the points of view of the social actors involved [4, 6].

Therefore, this qualitative synthesis aimed to synthesize, from the existing qualitative literature, the factors that influence women’s decision to participate in personalized breast cancer screening programs based on their individual risk, from the perspective of both women and health professionals. Also, to construct a conceptual model of the factors influencing women’s decisions. The results are expected to provide valuable information for actions to implement this strategy in different international contexts.

## Materials and methods

This systematic review of qualitative evidence was conducted according to the criteria in the Preferred Reporting Items for Systematic Review and Meta-Analyses (PRISMA) guidelines (Additional file 1). It was registered with PROSPERO under registration number CRD42022303159.

### Search strategy

We searched in six databases: Medline, Web of Science, Scopus, EMBASE, CINAHL, and PsycINFO. The search strategy, included terms related to “Breast cancer screening”, “Personalized risk assessment”, “Attitudes”, “Preferences” and “Decision making”. Boolean and wildcard search operators were used (Online Supplementary file 2). All publications reported up to March 30, 2022 were included.

One reviewer (CL) conducted the search in selected databases between January and March 2022 with no time limit. A manual search was also performed to identify additional studies using the Medline option “related articles”, and by relevant authors.

### Inclusion criteria

#### Setting

Breast cancer screening programs or hypothetical scenarios.

#### Population

(i) women participating in personalized breast cancer screening programs based on risk or asked about their preferences using hypothetical scenarios. (ii) HCPs from different disciplines and areas of work. We excluded studies involving women with breast cancer, and carriers of genetic variants of medium and high penetrance. We included studies involving subjects with cancer where results for breast cancer were presented separately.

#### Phenomenon of interest

Perceptions, attitudes, opinions regarding personalized breast cancer screening programs or factors influencing women’s participation.

#### Type of studies

(i) Qualitative studies of any design, using any qualitative technique for data collection and established qualitative data analysis techniques; (ii) mixed methods studies reporting qualitative findings separately from quantitative ones. We excluded books, opinion articles, case and review studies, conference proceedings, gray literature, and doctoral theses.

### Study selection

Two reviewers (CLV, MPV) independently evaluated titles and abstracts taking into account the previously established inclusion/exclusion criteria. Full-text versions of articles considered potentially relevant were obtained and reviewed. Disagreements were solved by discussion or consensus with the review team. We used Rayyan Intelligent Systematic Review software for study selection.

### Quality assessment

Two reviewers (CL, MP) independently assessed the quality using the Critical Appraisal Skills Program (CASP) [10] Discrepancies were resolved by discussion or consulting a third reviewer (ENDG) (Table 1).

**Table 1:** Critical Appraisal Skills Programme (CASP) evaluation of the studies included in the review of factors influencing participation in personalized breast cancer screening, until March 2022

No	Article	1	2	3	4	5	6	7	8	9	10
1	**29**	Yes	Yes	Yes	Yes	Can’t tell	No	Yes	Yes	Yes	NMC
2	**22**	Yes	Yes	Yes	Yes	Yes	No	Yes	Yes	Yes	NMC
3	**24**	Yes	Yes	Yes	Yes	No	No	Yes	Can’t tell	Can’t Tell	MC
4	**23**	Yes	Yes	Yes	Yes	Yes	Can’t tell	Yes	Can’t tell	Yes	NMC
5	**25**	Yes	Yes	Yes	Can’t tell	Yes	Yes	Yes	Can’t tell	Yes	NMC
6	**26**	Yes	Yes	Yes	Can’t tell	Yes	Yes	Yes	Can’t tell	Yes	NMC
7	**31**	Yes	Yes	Yes	Yes	Yes	Yes	Yes	Yes	Yes	NC
8	**18**	Yes	Yes	Yes	Can’t tell	Yes	Can’t tell	Yes	Yes	Yes	NMC
9	**29**	Yes	Yes	Yes	Yes	Yes	Yes	Yes	Yes	Yes	NC
10	**28**	Yes	Yes	Yes	Yes	Yes	Yes	Yes	Yes	Yes	NC
11	**32**	Yes	Yes	Yes	Yes	Can’t tell	No	Yes	Can’t tell	Yes	NMC
12	**33**	Yes	Yes	Yes	Yes	Yes	Can’t tell	Yes	Yes	Yes	NC
13	**27**	Yes	Yes	Yes	Yes	Yes	Yes	Yes	Can’t Tell	Yes	NC
14	**35**	Yes	Yes	Yes	Yes	Yes	Yes	Yes	Yes	Yes	NC
15	**36**	Yes	Yes	Yes	Yes	Yes	Yes	Yes	Yes	Yes	NC
16	**38**	Yes	Yes	Yes	Yes	Yes	No	Yes	Yes	Yes	NC
17	**34**	Yes	Yes	Yes	Yes	Yes	No	No	No	Can’t Tell	MC
18	**37**	Yes	Yes	Yes	Yes	Yes	No	Yes	No	No	MC

1. Was there a clear statement of the aims of the research?

2. Is a qualitative methodology appropriate?

3. Was the research design appropriate to address the aims of the research?

4. Was the recruitment strategy appropriate to the aims of the research?

5. Were the data collected in a way that addressed the research issue?

6. Was the relationship between researcher and participants adequately addressed?

7. Have ethical issues been taken into account?

8. Was the data analysis sufficiently rigorous?

9. Is there a clear statement of findings?

10. How valuable is the research?

*NC* No concerns, *NMC* No or very minor concerns, *MC* Moderate concerns

### Data extraction

We collected the following information:

i. Characteristics of the study: authors, year, country, and study context; objective, population, aspects of the method, whether it was part of a personalized screening study, and the main results.
ii. Information related to participants:
  Women: age range, race/ethnicity, nationality, socioeconomic status, educational level, occupation, and type of participation in personalized screening (actual, invited, and hypothetical).
  HCPs: professions, age, gender, and setting in which the study was conducted.
iii. Themes and findings of each study.

One reviewer (CL) conducted data extraction, and two reviewers (MP, EN) double checked the extracted information, that was discussed with the review team. We contacted authors in case of missing information.

### Data synthesis

We applied the “best fit’ framework synthesis approach”. This design is structured in an a priori framework for data extraction and analysis. It subsequently combines deductive and inductive analysis approaches [11]. Thus, evidence from included studies were coded against the themes of the a priori framework. New themes were generated from evidence that did not fit the a priori framework, leading to the development of a model to explain our phenomenon of interest [12].

We conducted a free search in MEDLINE for original articles and published reviews aimed to identify factors influencing women’s decision to participate in population-based personalized screening programs for breast cancer, and that used some type of conceptual and/or theoretical model. The terms used were Framework, Theory, Screening and Breast cancer.

We selected the *“Multilevel influences on the cancer care continuum*” (MICCC) model as the a priori framework. It postulates that health behavior is the product of seven levels of influence, and the experience of individuals with the health care system is influenced by these levels, and each one affects the others [13, 14]. This model was chosen because of its complexity, breadth of factors, and the possibility of analyzing the interrelation of the factors contained in it, as shown in previous studies [15–18].

We identified and organized the codes and themes mapped onto the seven levels of the MICCC model through deductive analysis and subsequently, locating themes that did not fit through inductive/interpretive analysis [19]. Thematic analysis was used to identify and analyze patterns (themes) within each level of the MICCC model including free coding, development of descriptive themes, and generation of analytical themes or third-order interpretations [20].

Two reviewers with expertise in personalized breast cancer screening and qualitative research (CL, MP) synthesized the first- and second-order data which were reviewed independently by two other reviewers (EN, MR). Reviewers reached a consensus on whether these corresponded with the pre-existing MICCC themes, or not (reciprocal translation process) [20].

The review team discussed preliminary themes organized in the MICCC model, and themes generated to be included inductively. One reviewer (CL) developed the analytical themes allowing progress from description to interpretation for the construction of a new more specific model to explain the factors influencing women’s participation in a personalized screening program.

### Reflexivity of the review team

During all stages of the process, the review team had a reflective stance, from the selection of the a priori framework to the synthesis of data and establishment of the final analytical themes. Our team had multidisciplinary backgrounds (Medicine, Nursing, Epidemiology, Statistics, and Public Health). Through the process, the progress was regularly discussed and decisions were made critically. Among included studies, one was published by the first author (CL). For this study the evaluation of methodological quality, and data extraction was performed by another reviewer (MP).

## Results

### Search results

**Fig. 1:**
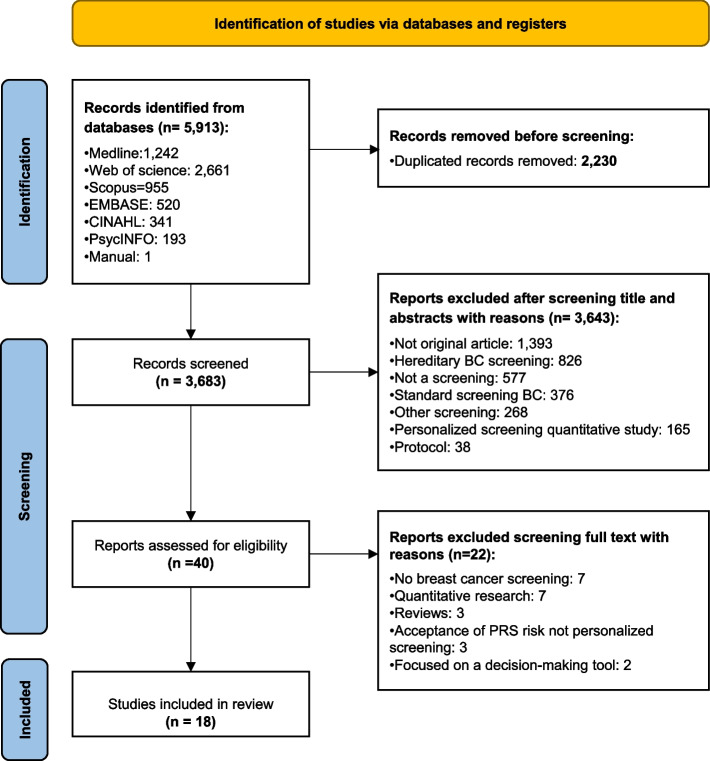
Preferred Reporting Items for Systematic Review and Meta-Analyses (PRISMA) flowchart of searches and documents included in the qualitative systematic review of factors influencing participation in personalized breast cancer screening, until March 2022

Articles that did not meet the criteria during screening were excluded with the reasons recorded in the PRISMA flowchart, which also reports the different phases of article selection [21] (Fig. 1).

### Characteristics of studies

Main characteristics of included studies are reported in Table 2 [18, 22–38]. Of the 18 studies, 17 were qualitative and one had a mixed methods design. No studies were excluded based on their quality.

**Table 2:** Characteristics of the studies included in the qualitative systematic review of factors influencing participation in personalized breast cancer screening, until March 2022

Authors/year/Country/setting	Aim	Participant characteristics	Method (Study design, sampling strategy and method of data collection and analysis)	BC personalized screening project
[28] UK; National Health Service Breast Screening Program	Explore prospective acceptability of possible risk-stratified breast screening (RSBS) scenarios in which screening frequency, age-range of eligibility and number of risk groups might vary.	25 women participants in screening with and without a family BC history	Qualitative Intentional Semi-structured interviews Thematic analysis	No
[23] UK; National Health Service Breast Screening Program	To elicit the views of women at low-risk about receiving this information, and their acceptability of less frequent breast cancer screening invites.	23 women at low-risk for breast cancer	Qualitative Intentional Semi-structured interviews Thematic analysis	BC-Predict
[38] Australia; The state breast screening program in Victoria and Parkville Family Cancer Center	To determine the acceptability of a to assign personalized BC risk assessments compared to standard current	31 women with and without a family history of breast cancer	Qualitative Intentional Focus groups interviews Questionnaire Thematic analysis	No
[22] UK; Areas of East Lancashire, London	To explore the views women and assess the acceptability have toward the implementation of risk stratified screening	19 British-Pakistani women from low socioeconomic	Qualitative Intentional Semi-structured interviews Thematic analysis	BC-Predict
[24] Netherlands, the United Kingdom, and Sweden; Studies on personalized screening: PRISMA, PROCAS and KARMA	To explore women’s perceptions of the implementation and organization of risk-based breast cancer screening and prevention.	143 women participating in three prospective cohort studies of personalized breast cancer screening	Qualitative Intentional Focus groups Questionnaire Thematic analysis	PRISMA, PROCAS and KARMA
[25] Netherlands, the United Kingdom, and Sweden; Studies on personalized screening: PRISMA, PROCAS and KARMA	To evaluate the adoption of risk‐based breast cancer screening and prevention by exploring perceptions of women.	143 women participating in three prospective cohort studies of personalized breast cancer screening	Qualitative Intentional Focus groups Thematic analysis	PRISMA, PROCAS and KARMA
[29] Australia; Victoria, LifePool cohort women	To explore breast screening participants’ views of the current program and examine their openness to change, and attitudes toward an individualized screening model.	52 women participants in the current breast cancer early detection program	Qualitative Intentional Focus groups Thematic analysis	No
[18] United States; New Hampshire and Vermont	To explore women’s views and personal acceptability of a potential risk-based mammography screening paradigm.	29 women residents of New Hampshire and Vermont	Qualitative For convenience Focus groups Questionnaire Content analysis and grounded theory	No
[37] United States; The Chicago Breast cancer in low-income primary care clinics	To explore the views on individual risk assessment of women of color and low-income women identified as being at increased risk of breast cancer.	13 Women high risk without previous history of breast cancer	Qualitative Intentional Semi-structured interviews Content analysis	No
[27] Spain; Primary Care Units in Barcelona and Lleida	To explore the barriers and facilitators of implementing a risk-based breast cancer screening program from the point of view of Healthcare professionals.	29 Healthcare professionals work area in relation to breast cancer	Socio-constructivist qualitative Theoretical Focus groups Thematic analysis	DECIDO
[30] UK; National Health Service Breast Screening Program	To elicit views regarding implementing less frequent screening for low-risk women from Healthcare professionals who implement risk-stratified screening.	28 Healthcare professionals work area in relation to breast cancer	Qualitative Intentional Focus groups Semi-structured interviews Thematic analysis	BC-Predict
[31] Canada; Breast cancer genetic counselling and screening services, Québec province	To explore Healthcare professionals’ perceptions of the application of a population-based approach to BC risk stratification.	15 Healthcare professionals directly involved in breast cancer genetic counselling or screening	Qualitative explorative Intentional and snowball sampling Semi-structured interviews Thematic analysis	PERSPECTIVE
[26] UK; National Health Service Breast Screening Program	To obtain the views of national health policy makers on the implementation of less frequent screening intervals for low-risk women.	17 Healthcare professionals directly or indirectly associated with the National Screening Committee and National Institute for Health and Care Excellence	Qualitative Intentional Semi-structured interviews Thematic analysis	BC-Predict
[33] Canada; Breast Screening Program, Quebec province	To examine equity in the delivery of services for the risk-based approach through stakeholder perceptions.	13 Professionals involved in the management, implementation or evaluation of the screening program	Qualitative Intentional and snowball sampling Semi-structured interviews Thematic analysis	PERSPECTIVE
[34] Canada; Centre Local de Services Communautaire, Montreal province	To explore the feasibility of any proposed implementation strategies for targeted breast cancer screening and to optimize communication tools for the risk stratification model	11 Healthcare professionals work area in relation to breast cancer	Qualitative descriptive Intentional Deliberative stakeholder consultations Thematic analysis	PERSPECTIVE
[36] Canada; Breast Screening Program, Quebec province	To explore the acceptability of different proposals for each step of women’s trajectory in the health care system in the event that a personalized approach.	20 Healthcare professionals work area in relation to breast cancer programs and in the policy	Qualitative descriptive Intentional and snowball sampling Semi-structured interviews Thematic analysis	PERSPECTIVE
[32] Germany; Gynaecology department of Munich Technical University; Munich	To assist doctors and screening participants in participatory decision-making.	15 physicians and professionals representatives of the public health service	Qualitative explorative Intentional Focus groups Content analysis	RISIKOLOTSE.DE
[35] Canada; Breast Screening Program, Quebec province	Analysis of decision-makers’ perspectives on genetic testing and insurance, as well as general insurability issues in the context of a risk stratification approach to breast cancer screening and prevention.	14 professionals’ decision-makers involved in the management of breast cancer screening programs and policies	Mixed design Intentional Semi-structured interviews Thematic analysis	PERSPECTIVE

All papers were published between 2017 and 2022, with the majority published in the last three years. Nine studies were conducted in Europe (five in the United Kingdom, one from Spain, one from Germany, and two multicenter studies conducted with women from the United Kingdom, the Netherlands and Sweden). Outside Europe, four studies were conducted in Canada, three in Australia and two in the United States. Eleven were nested in breast cancer personalization projects: Breast Cancer Predict and PRISMA (UK), PERSPECTIVE (Canada): DECIDO (Spain) and RISIKOLOTSE (Germany).

The models and factors included for risk prediction varied slightly across the different studies: the Decido study calculated the 5-year risk using The Breast Cancer Surveillance Consortium v2.0 (BCSC v2.0), which included age, race/ethnicity, first-degree family history of breast/ovarian cancer, personal history of benign breast disease, breast density, and PSR [7]. In PROCAS/BC-Predict, UK they used the Tyrer-Cuzick model (v8), which in addition to the abovementioned factors, took into account body mass index and height. In the PERSPECTIVE project, they calculated risk using the BOADICEA model, in which various lifestyle factors were added [4]. With the exception of the DECIDO project, the risk estimates were calculated at 10 years.

Nine studies included only women and aimed to explore the acceptability, views and perceptions of risk-based screening. Nine studies included HCPs and were focused on the exploration-description of determining aspects in the future implementation of personalized screening strategies. Eleven authors of selected studies were contacted to complete missing information. Three of them facilitated data, which accounted for five studies (Table 2).

### Characteristics of participants

Women: In total, 478 women participated in nine included studies. All, except one, included women under 50 years of age. Most women were from developed countries, white, and of medium–high socioeconomic and educational level. Two studies included women from ethnic minorities living in Western countries [22, 37].

In four studies [23–25, 37], women had participated in personalized screening programs: in one study, women were purposively selected who were estimated to be at low risk [23], in a second, only at high risk [37]. In the other two studies [24, 25], 34% were classified as high risk (Table 3).

**Table 3:** Characteristics of the women participating in the studies included in the qualitative systematic review of factors influencing participation in personalized breast cancer screening, until March 2022

Article	Age range	Race/ethnicity	Nationality	Socioeconomic status	Educational level	Occupation	Type of participation personalized Early Detection	Breast Cancer risk classification
[28]	Mean: 55 years 40–49 = 28% 50–59 = 36% 60–70 = 36%	White british: 76% asian: 16% black caribbean: 8%	British	High: 52% Medium–Low: 48%	University degree = 44% Non-university degree = 56%	Professional: 52% Non-professional: 48%	Hypothetical participation	NA
[23]	46–54: 69,5% 55–64: 13% 65–74: 17%	White british: 82% asian or asian british, indian, white european, black or black british, african, mixed (white & black Aafrican): 17%	British	High: 34.7% Medium–Low: 65.2%	University degree = 73,9% Non-university degree = 26,1%	Professional: 52% Non-professional: 48%	Participation	Only women at low-risk for breast cancer
[38]	Mean age 52 years 20–29: 6,4% 30–39: 9,6% 40–49: 29% 50–59: 19,3% 60–69: 32% 70 + = 3,2%	NR	Australian	NR	University degree = 41,9% Non-university degree = 58%	NR	Hypothetical participation	NA
[22]	Under 50 years of age: 26,3% Over 50 years of age: 63,1% Age not stated: 10,5%	Pakistani	Pakistanies	Only low	NR	NR	Hypothetical participation	NA
[24]	Range: 40 -74 years Netherlands: 57.5 [50-72] England: 56,0 [50-69] Sweden: 67 [44–76)	NR	Netherlands: 37,7% England: 35,55% Sweden: 26,5%	NR	NR	NR	Participation	Below average: 25% Medium: 25% Moderate: 15.8% High: 34.2%
[25]	Range: 40 -74 years Netherlands: 57.5 [50-72] England: 56,0 [50-69] Sweden: 67 [44–76	NR	Netherlands: 37,7% England: 35,55% Sweden: 26,5%	NR	NR	NR	Participation	Below average: 25% Medium: 25% Moderate: 15.8% High: 34.2%
[31]	Mean: 61 year Range: 48–72 years	Mostly White: 99%	Australian	Hight: 72,2% Medium–Low: 21,1% Missing: 5,8%	University degree = 66,6% Non-university degree = 38,5%	NR	Hypothetical participation	NA
[18]	Beetwen 40–74 years 50–74: 79.3% Less 49: 20.7%	White: 100%	American	NR	University degree = 82,3% Non-university degree = 79,3%	NR	Hypothetical participation	NA
[37]	18–39: 23% 40–49: 30,7% 50–69: 46%	Mostly black women african american: 85,6% latinas: 16,3%	American	Low	Non-university degree: 69%	NR	Participation	Only women at high risk of breast cancer

*NR* Not reported, *NA* Not applicable

HCPs: Overall, 162 HCPs participated in nine studies. Most were female (70%). The professional profiles were diverse, including physicians and nurses, policy makers of breast cancer screening, and Public Health programs. Three studies included genetic counselors and one included academic experts. The studies were performed in Primary Care contexts [3], and specialized centers of Breast Cancer Early Detection Programs [4] (Table 4).

**Table 4:** Characteristics of heath care professionals participating in the studies included in the qualitative systematic review of factors influencing participation in personalized breast cancer screening, until March 2022

Article	Professions	Age and gender	Study environment
[27]	Physician: 20,6% Management: 24,1% Nurse: 31% Specialists: 17,2% Psychologist: 7%	Age range: NR Women: 83,8% Men: 16%	Primary care University hospital Breast screening programs
[30]	Radiographer: 32,1% Advanced practitioner radiography: 17,8% General practitioner: 10,7% Consultant radiologist: 10,7% Cancer screening improvement lead: 7,1% Radiographer breast imaging manager, Superintendent radiographer/program manager, Breast screening office manager, Breast care nurse and Admin and data clerk: 17,8%	Age range: NR Women: 89,2% Men: 10,7%	Breast screening program Primary care
[31]	Physicians’ general practitioners: 40% physicians’ specialists: 30% Genetic counsellors: 30%	Age range: NR Women: 93,3% Men: 6,7%	Primary care University hospital Breast screening programs
[26]	Breast cancer HCPs (radiology, oncology, radiography, nursing and surgery): 35,2% Senior academics (ethics, epidemiology, statistics and health economics): 35,2% Breast screening program operations/management professions: 29,4%	Age range: NR Women: 64,7% Men: 35,2%	Breast screening program
[33]	Physician: 69,2% Nurse: 23% Lawyer: 7,6%	Age range: NR Male: 61,5% Female: 38,4%	Breast cancer screening program
[34]	Family physicians: 72.7% Genetic counsellors: 27,2%	Age range and gender: NR	Academic units Community health centers (Centre Local de Services Communautaires
[36]	Physician: 50% Nurse: 30% Other: 20%	Age range: NR Female: 65% Male: 35%	Cancer screening programs
[32]	Gynecologists: 46,6% General physicians: 13,3% Radiologists: 23,3% Genetic counsellors: 6,6% Public health service: 13,3%	Age range: NR Female: 60% Male: 40%	NR
[35]	Clinician/public health care: 14% Regional manager: 28% National manager: 21,4% Expert/public health care: 35,7%	Age range and gender: NR	Cancer screening programs

*NR* Not reported

### Themes

The findings of the studies were classified, synthesized and organized into major themes and sub-themes, generated from: i) deductive analysis of the contrast with the levels of the MICCC model, ii) inductive analysis of findings not considered in the MICCC model, from which three sub-themes emerged. In a first analysis, 6 themes, 21 sub-themes and 85 findings were identified; these were synthesized into 3 themes, 14 sub-themes and 43 findings (Table 5).

**Table 5:** Themes, findings, and authors of each study included in the review of factors influencing participation in personalized breast cancer screening, until March 2022

Themes	Sub themes	Findings	Authors
**1. Factors related to women**	**1.1 Beliefs about breast cancer, risk, and personalized early detection of breast cancer**	**HCPs** 1. Social perception of the susceptibility and severity of breast cancer **Women** 2. Fatalistic beliefs about breast cancer 3. Beliefs that explain the increased risk for breast cancer 4. Beliefs that explain the decrease in risk for breast cancer 5. Erroneous beliefs about screening personalization	1. Laza et al., 2022 [27]; McWilliams et al., 2020 [26] 2. Kelley-Jones, 2021 [28]; Anderson et al., 2018 [37] 3. Kelley-Jones, 2021 [28]; Sierra el al, 2021 [38]; Lippey et al., 2019 [29]; Anderson et al., 2018 [37]; McWilliams, et al. 2021 [23]; Woof et al., 2021 [30] 4. Kelley-Jones, 2021 [28]; McWilliams, et al. 2021 [23] 5. Kelley-Jones, 2021 [28]; He et al., 2018 [18]; Rainey et al., 2019 [25]
	**1.2 Knowledge on personalized early detection of breast cancer**	**Women** 6.General lack of knowledge about personalized screening strategies 7. Accurate knowledge about early detection 8. Women's reasons for knowing breast cancer risk **HCPs** 9. Negative consequences of lack of knowledge about personalized screening	6. He et al., 2018 [18]; Lippey et al., 2019 [29]; Kelley-Jones, 2021 [28]; Blouin-Bougie et al. 2021 [31]; Anderson et al., 2018 [37] 7. Anderson et al., 2018 [37]; Sierra el al, 2021 [38] 8. Anderson et al., 2018 [37]; McWilliams, et al. 2021 [23]; Sierra el al, 2021 [38]; Rainey et al., 2019 [25]; 9. Laza et al. 2022 [27]; Woof et al., 2021 [30]; McWilliams et al., 2020 [26]; Fürst et al., 2018 [32]
	**1.3 Psychological reactions to breast cancer risk estimation**	**Women** 10. Low risk generates a sense of relief and peace of mind 11. A high risk generates a feeling of anxiety and worry 12. Worrying about a high risk is unnecessary **HCPs** 13. Low risk generates anxiety and uneasiness 14. A high risk generates tranquility and decreases anxiety	10. McWilliams, et al. 2021 [23]; Rainey et al., 2019 [25] 11. Rainey et al., 2019 [25]; Blouin-Bougie et al., 2021 [31]; Anderson et al., 2018 [37] 12. Anderson et al., 2018 [37] 13. Woof et al., 2021 [30]; McWilliams et al., 2020 [26]; Levesque et al., 2019 [33] 14. Puzhko et al., 2019 [34]; Laza et al. 2022 [27]
	**1.4 Attitudes generated in the estimation of breast cancer risk**	**Women and HCPs** 15. Women who are estimated to be at low risk reject the recommendation to reduce screening intervals and opt for opportunistic screening 16. The estimation of a low risk generates non-attendance of women to screening tests 17. Women who are estimated to be at high risk accept the recommendation for more frequent screening and additional studies **HCPs** 18. Personalized screening generates women's proactivity in health care and participation in shared decision-making **Women** 19. The implementation of personalized screening generates altruistic attitudes in women	15. Kelley-Jones, 2021 [28]; Sierra el al, 2021 [38]; He et al., 2018 [18]; Lippey et al., 2019 [29]; McWilliams, et al. 2021 [23]; McWilliams et al., 2020 [26]; Laza et al. 2022 [27]; Woof et al., 2021 [30] 16. Lippey et al., 2019 [29]; Kelley-Jones, 2021 [28]; McWilliams et al., 2020 [26] 17. Kelley-Jones, 2021 [28]; Sierra el al, 2021 [38]; He et al., 2018 [18]; Lippey et al., 2019 [29], Woof et al., 2020 [22]; Anderson et al., 2018 [37]; Laza et al. 2022 [27]; Woof et al., 2021 [30] 18. Laza et al., 2022 [27]; Puzhko et al., 2019 [34] 19. McWilliams, et al. 2021 [23]; Kelley-Jones, 2021 [28]
	**1.5 Influence of other women's experiences**	**HCPs** 20. Other women's experiences of illness and death from breast cancer, messages from other women, and the number of possible risk-based pathways can cause confusion	20. McWilliams, et al. 2021 [23]; McWilliams et al., 2020 [26]; Woof et al., 2021 [30]; Laza et al., 2022 [27]; Puzhko et al., 2019 [34]
	**1.6 Health insurance coverage**	**Women** 21. Have health insurance coverage for personalized screening tests	21. He et al., 2018 [18]
**2. Factors related to personalized breast cancer screening strategies**	**2.1 Need for a change in the model for early detection of breast cancer**	**Women and HCPs** 22. Current model for early detection of breast cancer considered obsolete 23. Personalization is a logical step in the early detection of breast cancer 24. Implementation of personalization will allow revision of aspects of the current “one-size-fits-all” strategy	22. Kelley-Jones, 2021 [28]; Laza et al., 2022 [27]; McWilliams et al., 2020 [26] 23. Lippey et al., 2019 [29]; He et al., 2018 [18] 24. McWilliams et al., 2021 [23]; Sierra et al., 2021 [38]; Rainey et al., 2020 [24]
	**2.2 Advantages of personalized early detection of breast cancer**	**Women and HCPs** 25. Personalized screening is more cost-effective and efficient, and improves the quality of breast cancer detection and prevention services 26. Risk estimation provides valuable information for women's health care and for other women in the family 27. Reduced harms associated with screening for women at low risk 28. Earlier initiation and more frequent and prolonged screening for high-risk women	25. Sierra el al, 2021 [38]; McWilliams et al., 2020 [26]; Blouin-Bougie et al., 2021 [31] 26. Sierra el al, 2021 [38]; Kelley-Jones, 2021 [28]; Woof et al., 2020 [22]; Sierra el al, 2021 [38]; Anderson et al., 2018 [37] 27. He et al., 2018 [18]; Puzhko et al., 2019 [34]; Sierra et al., 2021 [38]; McWilliams et al., 2020 [26] 28. Laza et al., 2022 [27]; Blouin-Bougie et al., 2021 [31]; Fürst et al., 2018 [32]; Sierra et al., 2021 [38]; Rainey et al., 2020 [24]; Lippey et al., 2019 [29]; Anderson et al., 2018 [37]
	**2.3 Disadvantages of personalized early detection of breast cancer**	**Women** 29. Do not wish to change the current model in order not to lose the regularity of screening 30. Doubts about the scientific evidence supporting personalized screening	29. He et al., 2018 [18]; Kelley-Jones, 2021 [28]; Sierra el al, 2021 [38]; Rainey et al., 2019 [25]; McWilliams, et al. 2021 [23] 30. Rainey et al., 2019 [25]; He et al., 2018 [18]; McWilliams, et al. 2021 [23]; Kelley-Jones, 2021 [28]; Lippey et al., 2019 [29]; Woof et al., 2021 [30]
	**2.4 Women's need for information on personalized early detection of breast cancer**	**Women** 31. Inform women about the positive and negative aspects of personalized screening **Women and HCPs** 32. Development of educational actions for women by HCPs 33. Development of educational campaigns aimed at broad audiences through the mass media **HCPs** 34. Difficulties of HCPs in informing women with different barriers 35. Tools to improve understanding of women with different barriers	31. Kelley-Jones, 2021 [28]; Rainey et al., 2020 [24]; Lippey et al., 2019 [29]; McWilliams, et al. 2021 [23] 32. Blouin-Bougie et al. 2021 [31]; Rainey et al., 2019 [25]; He et al., 2018 [18]; Esquivel-Sada et al., 2019 [36]; Woof et al., 2021 [30]; Woof et al., 2021 [30]; McWilliams et al., 2020 [26] 33. Esquivel-Sada et al., 2019 [36]; Puzhko et al., 2019 [34] 34. Esquivel-Sada et al., 2019 [36]; Puzhko et al., 2019 [34]; Blouin-Bougie et al. 2021 [31]; Woof et al., 2021 [30] 35. McWilliams et al., 2021 [23]; Laza et al., 2022 [27]; Woof et al., 2021 [30]; McWilliams et al., 2020 [26]; Puzhko et al., 2019 [34]
	**2.5 Potential for inequity in access to personalized early detection of breast cancer**	**Women and HCPs** 36. The implementation of a personalized screening program could generate inequity in the access of women, especially those with various barriers	36. Woof et al., 2021 [30]; Blouin-Bougie et al. 2021 [31]; Blouin-Bougie et al. 2021 [31]; Puzhko et al., 2019 [34]; Levesque et al., 2019 [33]; Rainey et al., 2020 [24]
	**2.6** **Potential genetic discrimination of personalized early detection of breast cancer**	**Women and HCPs** 37. The implementation of a personalized screening program could lead to genetic discrimination of women at high risk	37. Sierra el al, 2021 [38]; Lippey et al., 2019 [29]; Levesque et al., 2019 [33]; Dalpé et al., 2017 [35]
**3 Factors related to HCPs**	**3.1 Lack of knowledge of HCPs**	**Women and HCPs** 38. HCPs do not have sufficient knowledge and training on personalized screening and genetic issues 39. HCPs do not have adequate communication skills **HCPs** 40. The need for collaboration between family physicians and geneticists 41. Experiences of health care HCPs in risk communication of other early detection programs	38. Rainey et al., 2020 [24]; Laza et al., 2022 [27]; Puzhko et al., 2019 [34] 39. Kelley-Jones, 2021 [28]; McWilliams, et al. 2021 [23]; Levesque et al., 2019 [33]; Laza et al., 2022 [27]; Blouin-Bougie et al. 2021 [31]; Puzhko et al., 2019 [34]; Fürst et al., 2018 [32] 40. Blouin-Bougie et al. 2021 [31] 41. Levesque et al., 2019 [33]; Laza et al., 2022 [27]; McWilliams et al., 2020 [26]
	**3.2 Need to support women in decision making**	**Women and HCPs** 42. The need for support from HCPs for women in decision making 43. Women's closeness and trusting relationship with primary care professionals	42. Kelley-Jones, 2021 [28]; McWilliams, et al. 2021 [23]; Rainey et al., 2019 [25]; Laza et al., 2022 [27]; Woof et al., 2021 [30]; McWilliams et al., 2020 [26]; Laza et al., 2022 [27]; Levesque et al., 2019 [33] 43. Laza et al., 2022 [27]; Woof et al., 2021 [30]; McWilliams, et al. 2021 [23]

*HCPs*: Health Care Professionals

**Fig. 2:**
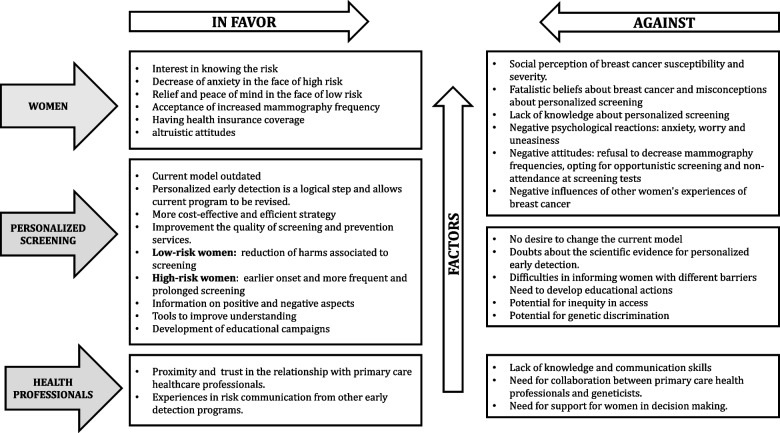
Factors for and against women’s participation in personalized breast cancer screening programs obtained from the systematic review

The results are presented through a model showing the factors for and against women’s participation in personalized breast cancer screening programs. We also included the perspectives and opinions of HCPs on these factors. The opinions of HCPs are derived from the relationships established with women who have participated and/or allegedly participated in a personalized screening program (Fig. 2).

### Theme 1. Factors related to women

#### Beliefs about breast cancer, risk, and personalized early detection of breast cancer

HCPs reported a strong social perception of women’s susceptibility and severity of breast cancer [26, 27]. They identified fatalistic beliefs expressed by women: it is a common and “omnipresent” disease, which appears randomly [28], without reason or pattern, and cannot be avoided. Therefore, all women are at risk and vulnerable to suffer from it no matter what they do to avoid it [37].

Some beliefs associated to an increased risk of breast cancer included: having a family history of breast cancer [23, 28, 29, 37, 38], large breasts, not having children [23, 30, 37], have undergone in vitro fertilization treatments [24, 28], being in poor health, and lifestyle factors (unhealthy diet and smoking) [37]. On the other hand, it is believed that the risk decreases with increasing age and menopause [26, 28], no family history of breast cancer in the first line of con-sanguinity, and healthy lifestyle behaviors [28]. In relation to personalized screening, it was believed that more screening tests were better and fewer tests allowed early diagnosis to be missed [18, 28], genetic testing could accurately predict a diagnosis and when there is a family history, the onset of the disease skipped a generation [25].

#### Knowledge on personalized early detection of breast cancer

Women’s knowledge of personalized screening showed mixed results, overall, a low general knowledge prevailed [18]. Genetic testing was considered strange, an incomplete, unknown, or future science, rather than a currently useful information and technology. There was concern about the overemphasis on genetics in the algorithm for risk calculation [30]. Personalized screening was considered a diagnostic procedure and ongoing risk estimation was necessary because of the modifiable nature of breast cancer and some lifestyle-related risk factors (body mass index, alcohol consumption, physical activity); and others such as the use of hormonal contraceptives [28, 31, 37]. In general, there was not sufficient knowledge and understanding of the risk and the probabilities of developing breast cancer according to the estimated risk [37].

HCPs considered that lack of knowledge had negative consequences by making it difficult to understand the benefits of personalization [27], increasing the chances of misinterpreting risk (low risk as no risk) [23, 26], and overestimating and/or denying it [32].

Two studies reported that women had adequate knowledge of this strategy, in a context where they were aware of risk due to family history of breast cancer: early detection decreased the likelihood of breast cancer being fatal [37], and risk estimation could save the lives of women at high risk and those with breast cancer family history offering early detection and prevention [29].

Reasons women reported to know their risk included: (i) general curiosity, knowledge is considered “power”; (ii) to learn more about breast cancer, be aware of the disease and detect it early; (iii) to help future generations (daughters and young women in the family) in the early detection of the disease [37]; (iv) to decide on the use of hormone replacement therapy during menopause [23]; (v) to accept screening frequency recommendations [25, 29, 37].

#### Psychological reactions to breast cancer risk estimation

Psychological reactions were not homogeneous and vary according to the real or hypothetical estimation of breast cancer risk.

In two studies where women participated in a personalized early detection program, the estimation of a low risk produced a sense of relief and peace of mind, as they did not consider the disease a direct and immediate threat to their lives and could thus reduce the frequency of screening [23, 25]. However, in another group, knowing that they were at low risk did not have much impact [23, 25]. In contrast, HCPs felt that a low risk generated anxiety and uneasiness due to the change in screening strategy [30], with longer mammography intervals and fear of a late stage diagnosis [26, 33].

A high risk also generated anxiety and uneasiness, but for other reasons: the perception of breast cancer as a burden in women’s lives related to the feelings of guilt and stigma generated by the disease [25]. It also caused helplessness and fear by feeling that cancer is *“inevitable”* [25, 31, 38]. Likewise, because of the generation of contradictory feelings: at the same time that they offload on themselves the responsibility for their breast health, it is not their responsibility if they develop breast cancer [25].

However, for other women worrying about high risk was considered unnecessary because it did not mean they would develop breast cancer, and they only needed to be diligent with early detection of the disease. Also, because having a family history of chronic diseases, such as diabetes and hypertension, were perceived as more immediate threats that diminished concern for high risk of breast cancer [37].

HCPs did not agree with women on the negative impact that a high risk could have, stating that it could be reassuring and decrease anxiety, especially for those who have a reason to worry about a diagnosis of breast cancer [27, 34]. However, HCPs in a Canadian study considered that a factor generating anxiety would be the possibility that women at high risk could be treated differently by health insurers [33, 35].

#### Attitudes generated in the estimation of breast cancer risk

As with psychological reactions, the attitudes generated are heterogeneous and depend on the estimation and/or the risk category.

A low risk generated women’s rejection of the recommendation to expand the screening intervals, opting for opportunistic screening to maintain a higher frequency of detection [18, 28, 29, 38]. This is explained by the belief that *“more screening is better”*, which becomes a source of reassurance and a greater perceived control, considering the negative experiences of other women who have had breast cancer [28]. Also, because of the fear of missing a timely diagnosis, which outweighs the discomfort of a false positive result [18]. For this reason, they questioned that longer screening intervals respond to the interest of the health system to reduce costs [23]. At the same time, in other women it generated attitudes such as the decision not to attend screening tests due to a false *“sense of security”* [26, 28, 29].

The estimation of a high risk led to the acceptance of recommendations for more frequent screening and further studies [18, 28, 29, 38], so they would continue to attend screening mammography [26, 28, 37] as it would allow them to have a greater surveillance of their health [38].

Despite agreeing with the attitudes described by women [23, 27], HCPs also considered that the implementation of personalized screening strategies could produce other positive attitudes in women, such as proactivity in health care, given the growing interest in decision making related to their health [27]; and the increased participation in shared decision making [34].

A novel finding identified in two studies is female altruistic attitudes, stating that longer screening intervals for women at low risk would allow more testing to be offered to those at high risk; giving them the opportunity to detect and treat breast cancer early, and allowing for a reasonable allocation of health system resources [23, 28].

#### Influence of other women’s experiences

Several HCPs perceived that for women, known experiences of breast cancer disease and breast cancer death [23], and the messages received from other women in the family and/or environment, as well as the number of possible risk-based pathways [26]may cause confusion and discourage participation in personalized screening [23, 30]; particularly in young women with children [26] and for those with a family history of breast cancer [26, 27, 34].

#### Health insurance coverage

In a U.S. study, women reported that participation is enhanced if risk estimation and more frequent screening tests are covered by their health insurance [18].

### Theme 2. Factors related to personalized breast cancer screening strategies

#### Need for a change in the model for early detection of breast cancer

Two studies, one involving women [28] and another HCPs [27], agreed that the current model of care for breast cancer screening was considered outdated as it was not in line with both new medical technologies and genetic profiling for the calculation of individual risk. They considered that women have a different risk from one another [26] so that personalization is seen as the “logical step” that allows individualizing early detection [18, 29]. Likewise, implementation will make it possible to review aspects of current population-based screening programs, such as the age of initiation and completion of screening [23, 24, 38].

#### Advantages of personalized early detection of breast cancer

Both groups agreed that personalized early detection has major advantages for health systems compared to the current “one-size-fits-all” strategy: it is more economical and efficient [37, 38] and improves the quality of breast cancer early detection and prevention services, through: i) greater accuracy of risk assessment, ii) identification of women at risk, and iii) personalization of management and follow-up of women [31].

On the other hand, risk estimation provides valuable information to all women, especially those who may develop breast cancer at a younger age, and those with no family history of breast cancer [29]. It would also help HCPs to monitor breast health more effectively [22, 26], which is beneficial not only for them, but also for other women in their families, such as daughters [37, 38].

For low-risk women, it reduces the harms associated with screening (over-diagnosis and false-positive results) [18, 34], and the number of mammograms and additional tests [38], which implies less inconvenience and discomfort [22]. For high-risk women, it makes it possible to assess and begin early detection before the age of 50, a more frequent and prolonged screening, and the addition of other tests such as ultrasound or magnetic resonance imaging [27, 31, 32, 38]. It also makes it possible to modify lifestyles at a younger age [25]. Therefore, it detects malignant lesions earlier [29], increases the chance of survival of diagnosed women, helps to guide future decision making, and the chance to be referred to preventive services [37].

#### Disadvantages of personalized early detection of breast cancer

Despite the advantages expressed, in three studies, some women did not wish to change the current model, considering that they would lose the regularity and security it offers them. [18, 28, 38]. Thus, they suggested that participation in personalized screening should be optional [28], voluntary [25], and the increase in the frequency of screening should not be drastic, since women after the age of 50 experience health and physical changes due to menopause [23].

Another aspect against are the doubts generated among women about the scientific evidence supporting personalized screening, such as the accuracy in the estimation of risk, the criteria on which the weighting of the different risk factors is made, their calculation, and the establishment of screening intervals [18, 23, 25, 28]. There is concern that a change to personalized screening might be driven by the saving of financial resources of the health systems, and not by its benefits [18, 25, 29, 30]. This view is due to negative experiences of modifications in health policies [23, 29].

#### Women’s need for information on personalized early detection of breast cancer

One factor to which women draw attention is that they should be given clear and concise information about the positive and negative aspects of personalized screening and the changes in the screening model [24, 26, 29]. They consider one key aspect: showing the evidence that supports reducing the frequency of screening for women at low risk [23].

For the latter issue, both groups considered essential the development of educational actions by HCPs [31], to inform about the scientific basis of a personalized model and its recommendations [18, 25], emphasizing the risk assessment and the characteristics of the approach [36], of a better benefits/harm balance for all risk groups, and of the modifications in the frequency of early detection tests [30]. Two issues were considered fundamental: informing that low risk does not mean having *“no risk”* or an *“immunity”* for breast cancer [26, 30], and that the move to personalization does not respond to a policy of *“cost reduction”* [23].

However, HCPs referred to the difficulties in informing women. In particular they referred to barriers such as low health literacy, language, cultural and religious barriers, given how complex it can be for them to understand the aspects related to genetics and personalized screening [34, 36]. Faced with this drawback, they suggested two actions: the use of graphic, written, online tools, risk/benefit calculators; as well as verbal explanations in personal and/or virtual meetings to improve communication and understanding of women [30, 31, 34, 36].

Likewise, the development of educational campaigns aimed at broad audiences through the mass media, which raise awareness in society, reduce resistance to change, and facilitate the participation of women in personalized screening [23, 27, 31, 34]. Regarding the mass media, they call attention to the importance of clear and coherent information because confusing and distorted messages can negatively affect women’s understanding and participation [30], and the credibility of the future program [22].

#### Potential for inequity in access to personalized early detection of breast cancer

Canadian HCPs expressed two factors that could lead to inequity in women’s access to a personalized screening program [30, 31]: it would enhance the current resource limitations of the health system in the face of increased demand for early detection tests [23, 31]; especially for those without primary care providers [34] and those residing in rural and/or remote areas [36]. Second, the difficulties for women with low literacy and educational levels, and migrants with cultural and language specificities to understand and discuss the concepts of genomics and risk, and to make informed participation decisions given the complexity that personalization adds to informed consent [36].

In the United Kingdom, several women and HCPs expressed a similar concern in resources limitations, but related to the costs of mammograms and additional examinations [24].

#### Potential genetic discrimination of personalized early detection of breast cancer

In Canada and Australia, both groups reported that the estimation of a high risk may be considered a pre-existing condition by health insurers and it may affect this group of women in their health coverage, in obtaining life and health insurance, and in their employability [29, 35, 36, 38]. Therefore, HCPs suggest that it is necessary to disclose to women the potential impact on insurability before undergoing genetic testing, and to generate a public debate on access to genetic information by health insurers, as well as to limit and/or prevent its access and use in medical records [35].

### Theme 3. Factors related to health care professionals

#### Lack of knowledge of health care professionals

Women and HCPs themselves consider that HCPs do not have sufficient knowledge and training on personalized screening and genetic issues [25, 27, 34], or adequate communication skills [23, 28]. They considered these aspects as fundamental to inform, advise and guide women in making informed decisions to participate in personalized screening and accept the recommendations [23, 27, 28, 31, 32, 34, 36].

Likewise, HCPs stated that in addition to training, collaboration between primary HCPs and geneticists is essential [31]. In this regard, they highlighted as an aspect in their favor, that they have experience in risk communication in other early detection programs such as prenatal, cervical and prostate cancer [22, 27, 33].

#### Need to support women in decision making

Both groups stated that women need the support of HCPs to be more confident in their decision making, as they may feel overwhelmed by the complexity of information and weighing the pros and cons of participating in personalized screening [22, 25, 27, 28, 30]; especially for those who will have to decrease screening frequency [22], and those with low educational level and/or language barriers [23, 27, 36].

A positive aspect reported by HCPs is the closeness and trusting relationship between women and their primary care professionals [23, 27]. However, they also reflect that for some women the decision to participate and accept the recommendations is a passive one, as they feel unable to do so on their own and trust the professionals to decide what is best for their health [23, 30].

## Discussion

The qualitative synthesis describes the factors that influence women’s decision to participate in personalized breast cancer screening programs, from the perspective of women and HCPs. Its results show the novelty of the research (the first studies are less than 10 years old), and a growing interest in the topic.

This inventory of factors in favor and against shows a balance. The most important factors in favor were those related to the implementation of personalized breast cancer screening, and against, those of women themselves.

### Main factors for and against

The fact that the factors favoring participation are focused on those specific to personalized screening is encouraging and highlights its good general acceptance [39–42]. Also, it relates to an increased societal awareness of risk stratification by improving the harms of age-based screening [43]. The results of the synthesis show that there is a consensus that personalization is a positive and a necessary progression from the current age-based approach to population screening [42, 44].

Despite the acceptance of the strategy, women’s own factors that do not favor participation show a greater weight among all the findings identified. Aspects such as low knowledge about the strategy, fatalistic beliefs about breast cancer, negative influences from other women, and negative psychological reactions and attitudes towards risk estimation are consistent with other studies on breast cancer personalization [39, 45, 46], and have been widely documented as strong barriers to participation breast cancer screening [47–49], particularly for women with low health literacy [50]. Therefore, these factors could be thought of as *“inherited”* from the current *“one-size-fits-all”* model of early detection.

### Psychological effects

Special attention should be paid to the psychological reactions and negative attitudes generated by risk estimation. A review by Vallone et al. [51] reinforces the idea that risk estimation is connected with the social perception of breast cancer. Early detection generates fears in women for a possible positive result, producing two dichotomous responses in women who perceive a high susceptibility to breast cancer: tranquility (greater participation) versus avoidance (defensive attitude).

However, and contrary to the results of the synthesis, in the case of personalization, three randomized trials conducted with women who have had actual participation in personalization strategies show that there are no significant differences in the anxiety status, neither in the attitudes across women who are estimated at high risk and those who are not [52–54]. Two recent studies showed a high acceptance of personalized screening recommendations, regardless of the estimated risk [55, 56].

On the other hand, HCPs involved in the BC-Predict project, argue that personalized screening provides a well-defined pathway (increased screening frequency, additional procedures and prevention options) for women at high risk, which could minimize anxiety [44]. Some women would feel help to decrease anxiety by a clear communication of individual risk results, establishing a follow-up for personalized assessment, and providing practical steps to manage it [56].

### Aspects for implementation

Thinking about a future implementation of personalized breast cancer screening involves an analysis of the abovementioned female factors and focusing the work on them. Several aspects to reduce the weight they carry are those provided by the results of this synthesis as facilitating factors for participation. On the one hand, the relationships of trust built between women and their primary HCPs is essential [57, 58]. On the other hand, the experiences and lessons learned by professionals in current early detection programs, such as regular interactions with women, making clear and consistent recommendations, having enough time to listen to them, and using other individual contacts (text messages, reminder letters, and motivational calls). In addition, the use of inclusive language and behaviors with LGTBI women, those with language and cultural barriers, and care by female professionals to avoid women feeling embarrassed [51].

### Tools to improve understanding

Other positive factor that emerged from the results is the use of tools to improve women’s understanding of personalization and to support practitioners in doing so. Several studies conducted in the settings of the WISDOM, PROCAS and PERSPECTIVE projects showed their usefulness [59–61]. In the former, 93% of low-literacy women reported as very useful an interactive virtual tool for risk assessment, helping to understand the risk and likelihood of developing breast cancer [59]. In the PROCAS cohort, an informative booklet to facilitate informed decision making showed to improve understanding of genomic testing in more than 50% of participants [60]. The PERSPECTIVE electronic platform revealed a significant increase in generating changes in knowledge, understanding, and the interest in in genetic testing [61].

### Information and communication

The task of informing and educating women is negatively affected by the low knowledge of HCPs on issues of genetics and screening personalization. A recent qualitative synthesis identified that this is one of the most important issues to consider in order to implement personalized screening [62]. It is essential to emphasize further training of HCPs to assess, interpret and communicate risk [63]. Lapointe et al. propose that the training programs of future professionals should include more content on genetic issues and continuous professional training to address these knowledge gaps [64]; along with the generation of clinical guidelines and protocols for the implementation of personalized breast cancer screening [65]. Along with additional training, another key aspect is the acquisition of communication skills so that HCPs can confidently navigate complex genetic and personalized screening issues [66]. These acquired knowledge could play a key role in informing women and enabling decision making about the potential participation in personalized screening, and in the acceptance of the recommendations emanating from this process. Another facilitator are the contributions that can be achieved by the development of educational campaigns in the mass media, which would not only have an impact on women, but also on society in general. Mass media campaigns have become powerful instrument to inform, sensitize and raise awareness about the current breast cancer screening, showing excellent results in increasing interest and knowledge, and decreasing fears and reluctance to perform screening mammograms [67–69]. However, it is important to consider that the mass media must have clear and consistent information about personalized screening. This last is key, because the information disseminated is not always accurate [70] and journalists do not always have solid knowledge to analyze medical evidence, producing an information bias [71].

The development of mass media campaigns should also include other elements. First, the extensive experience in public education of social and patient organizations [72]. Second, there need for a feminist agenda that generates female empowerment, thought of as a dynamic process to empower women and promote their autonomy in decision-making. A process that helps to control their health and well-being, and to ask questions and demand resources from their political representatives [73]. The process should prioritize women’s points of view, knowledge and experiences as valid forms of knowledge; the rights over the body and its care. It should also allow the construction of trust and to be inclusive, allowing the gathering and respect for female diversity [74, 75].

### Inequity

Finally, two negative findings that should not be overlooked, although they were only referenced in non-European contexts: the possibility that the implementation of personalized breast cancer screening strategies may generate inequity in women’s access to early detection, and the genetic discrimination that health insurers may establish against women with a high risk of breast cancer. This issue is relevant due to the worldwide imposition of reforms emanating from neoliberal policies in health systems, which have led to privatization, decentralization and fragmentation of health systems, as well as to a decrease in universal access of the population [76, 77].

### Limitations and strengths

Among the limitations stands out the inclusion of studies conducted in high-income and mostly European countries. Nevertheless, several aspects strengthen the findings of the study: the heterogeneity of the sociodemographic characteristics of women and the plurality of disciplines of the professionals participating in the studies. In addition, half of the primary studies were nested within the large breast cancer personalized screening projects conducted or currently underway, implying that participants may have had more knowledge about personalized strategies. As strengths, this is to our knowledge, the first review that comprehensively identified and described the factors, for and against, influencing women’s participation in personalized breast cancer screening. the review includes the points of view of HCPs from their professional activity. It also includes all the studies on the subject carried out to date, without time limitation. The use of “The Best fit” framework synthesis design allowed the creation of a new specific framework to understand the study phenomenon, starting from the existing one that explains the continuum of care process for breast cancer.

## Conclusions

The synthesis provides, the pro and con factors that influence women’s participation in personalized breast cancer screening programs. This information is of interest for future implementation of personalized screening programs. As a strong factor emerges the characteristics of the personalized screening strategy, and women’s own factors emerge as a negative aspect. Future implementation requires women’s literacy processes, as well as qualification processes for HCPs in genetics and personalized screening, and to improve their communication skills.

### Supplementary Information

**Additional file 1.** PRISMA for systematic review protocols (PRISMA-P) 2015 checklist for this systematic review.

**Additional file 2. **Search string.
