# Supplementary material for: “For and against” factors influencing participation in personalized breast cancer screening programs: a qualitative systematic review until March 2022
Source: Arch Public Health. 2024 Feb 22;82:23. doi: 10.1186/s13690-024-01248-x (PMC10882761; doi:10.1186/s13690-024-01248-x)
Supplement: Supplementary file 2 — Additional file 2. Search string. [file 13690_2024_1248_MOESM2_ESM.docx]

**Additional file 2. Search string**

| **Topic** | **Terms and results** | **No.** |
| --- | --- | --- |
| **Breast cancer screening** | | |
| #1 | "Breast Neoplasms"[Mesh] | 316,353 |
| #2 | Breast[tiab] | 473,416 |
| #3 | #1 OR #2 | 515,331 |
| #4 | "Mass Screening"[Mesh] | 137,424 |
| #5 | "Early Detection of Cancer"[Mesh] | 31,284 |
| #6 | screen*[tiab] | 851,607 |
| #7 | #4 OR #5 OR #6 | 902,478 |
| #8 | #3 AND #7 | 43,504 |
| #9 | "Mammography"[Mesh] | 31,646 |
| #10 | Mammogra*[tiab] | 34, 484 |
| #11 | #8 OR #9 OR #10 | 68,786 |
| **Personalised risk assessment** | | |
| #12 | "Risk assess*"[tiab] | 82,601 |
| #13 | "Risk stratifi*"[tiab] | 38,627 |
| #14 | "Personalized risk"[tiab] | 547 |
| #15 | "Personalized risk"[tiab] | 90 |
| #16 | "Risk-based"[tiab] | 7,357 |
| #17 | "Risk prediction"[tiab] | 12,557 |
| #18 | "Snp testing"[tiab] | 47 |
| #19 | “Single Nucleotide polymorphisms” [tiab] | 58,642 |
| #20 | "Polymorphism testing"[tiab] | 43 |
| #21 | "Genetic testing"[tiab] | 25,760 |
| #22 | "Polygenic risk scores"[tiab] | 1194 |
| #23 | "Precision medicine"[tiab]] | 14,582 |
| #24 | "Susceptibility genes"[tiab] | 7,582 |
| #25 | #12 OR #13 OR #14 OR #15 OR #16 OR #17 OR #18 OR #19 OR #20 OR #21 OR #22 OR #23 OR #24 | 238,043 |
| **Attitudes, Preferences and Decisión making** | | |
| #26 | “Decision making"[MeSH] | 218,380 |
| #27 | "Decision*"[tiab] | 436,902 |
| #28 | "Attitude*"[tiab] | 170,347 |
| #29 | "Attitude"[MeSH] | 613,726 |
| #30 | "Opinion*"[tiab] | 114,534 |
| #31 | "Viewpoint*"[tiab] | 39,220 |
| #32 | "Belie*"[tiab] | 321,953 |
| #33 | "Perception*"[tiab] | 295,024 |
| #34 | "Perception"[MeSH] | 459,664 |
| #35 | "Experience*"[[tiab] | 1,200,442 |
| #36 | "Willingness"[tiab] | 30,565 |
| #37 | "Barrier*"[tiab] | 344,271 |
| #38 | "Facilitator*"[tiab] | 31,289 |
| #39 | "Participat*"[[tiab] | 600,834 |
| #40 | "Patient participation"[MeSH] | 28,032 |
| #41 | "Knowledge*"[[tiab] | 806,063 |
| #42 | "Intention*"[tiab] | 97,479 |
| #43 | "Intention"[MeSH] | 13,677 |
| #44 | "Benefit*"[tiab] | 767,208 |
| #45 | "Preference*"[tiab] | 171,142 |
| #46 | "Understand*"[tiab] | 1,343,025 |
| #47 | #26 OR #27 OR #28 OR #29 OR #30 OR #31 OR #32 OR #33 OR #34 OR #35 OR #36 OR #37 OR #38 OR #39 OR #40 OR #41 OR #42 OR #43 OR #44 OR #45 OR #46 | 5,855,401 |
